# Diverging trends in erythrocyte size elucidate cardiovascular evolution in stem dinosaurs and crocodilians

**DOI:** 10.1098/rspb.2025.1286

**Published:** 2025-09-10

**Authors:** Paul Joseph Byrne, Lucas J. Legendre, Scott Echols, Colleen G. Farmer, Yun-Hsin Wu, Adam K. Huttenlocker

**Affiliations:** ^1^ Department of Earth Sciences, University of Southern California, Los Angeles, CA, USA; ^2^ Dinosaur Institute, Natural History Museum of Los Angeles County, Los Angeles, CA, USA; ^3^ Department of Earth and Planetary Sciences, The University of Texas at Austin, Austin, TX, USA; ^4^ Medical Center for Birds, Oakley, CA, USA; ^5^ Department of Biology, University of Utah, Salt Lake City, UT, USA; ^6^ Department of Zoology, University of Dublin Trinity College, Dublin, Ireland; ^7^ Division of Integrative Anatomical Sciences, University of Southern California Keck School of Medicine, Los Angeles, CA, USA

**Keywords:** erythrocyte, Pseudosuchia, Avemetatarsalia, phylogenetic eigenvector maps, palaeohistology, cardiovascular evolution

## Abstract

Red blood cell (RBC) size constrains the rate of diffusion of gases between (i) the environment and the capillary beds of the gas exchanger and (ii) the blood and organs. In birds, small RBCs with a high surface area to volume ratio permit a high O_2_ diffusion capacity and facilitate sustained, vigorous exercise. Unfortunately, our knowledge of archosaur cardiovascular evolution is incomplete without fossilized RBCs and blood vessels. However, muscle capillary diameters closely match RBC width and, importantly, these microvessels leave a signature in bone in the fossil record. Here, we ask: do fossilized, histological indicators of RBC size, combined with phylogenetic information, support divergent patterns of cardiovascular evolution in Mesozoic crocodile-line and bird-line archosaurs? Building on a published dataset, we used vasculo-lacunar histometrics and phylogeny to retrodict RBC sizes in 20 extinct and 20 extant tetrapods. Our results indicate decreases in RBC size within the archosauromorph *Prolacerta* and in bird-line archosaurs (Avemetatarsalia). Conversely, crocodile-line archosaurs (Pseudosuchia) that transitioned to an aquatic environment demonstrated increases in RBC size. These patterns offer an opportunity to probe physiological hypotheses regarding archosaur cardiovascular evolution and can explain, in part, the contrasting aerobic capacities of extant species in these two major archosaur lineages.

## 1. Introduction

The two major extant lineages of archosaurs—crocodilians and birds—exhibit remarkable differences in their physiology and activity patterns governed by their respective ectothermic and endothermic lifestyles. Famously, the cardiovascular systems of these taxa reflect their divergent evolutionary pathways, with crocodilians possessing a shunting mechanism that optimizes energy efficiency in low-oxygen environments, whereas birds have a fully divided four-chambered heart that supports their high metabolic demands [1,2]. Although there has been considerably less research on this topic, crocodilians and birds exhibit extraordinary variation in the size and volume of their red blood cells (RBCs), which constrain the bulk transport and diffusion of gases to and from bodily tissues [3].

In birds, small RBC size contributes to shorter diffusion distances and permits fast O_2_ uptake kinetics [3–7], and has been shown to be smallest in volant species compared with divers and aquatic species [8]. This increased uptake kinetics results in an elevated energetic capacity. On the other hand, crocodilians exhibit relatively large RBCs within vertebrates [3] and do not have short diffusion distances. As a result, most crocodilians cannot permit sustained, vigorous exercise and have relatively low O_2_ uptake kinetics.

In the past decade, there has been substantial palaeobiological research into understanding the origin of high metabolic rates and elevated energetics within tetrapods, especially among archosaurs and synapsids [9–17]. At the backbone of this emerging field, bone histology has proved to be a robust method capable of generating quantifiable data to infer physiological features within fossil vertebrates. It has been recognized that changes in bone microvasculature can be used as a proxy for RBC size, as capillary dimensions constrain the maximum size of the RBC [3,10]. In this study, we predicted RBC size using bone histometrics to examine trends in energetic capacity at the divergence between bird-line and croc-line archosaurs. Extant archosaurs include birds and crocodilians, two groups that exhibit vastly different energetic, metabolic and behavioural profiles. Reconstructing RBC size evolution at the divergence between these two groups can shed light on the evolution of modern crocodilian and bird physiology and can generate hypotheses regarding their persistence and diversification through past and present global abiotic crises.

## 2. Results

### (a) Bone histometrics, phylogeny and red blood cell size

**Figure 1.**
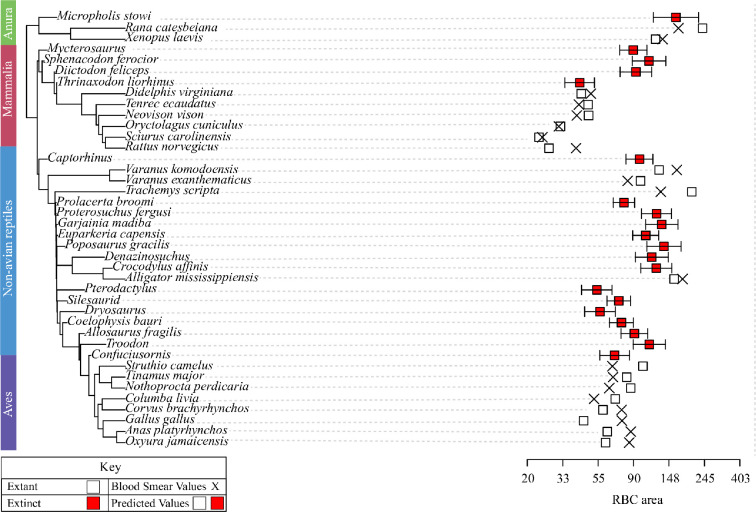
PEM showing raw and predicted values for RBC_area_. Notable increases in RBC_area_ are prevalent in croc-line (Pseudosuchia) archosaurs, and significant decreases in RBC_area_ occur within bird-line (Avemetatarsalia) archosaurs.

**Figure 2.**
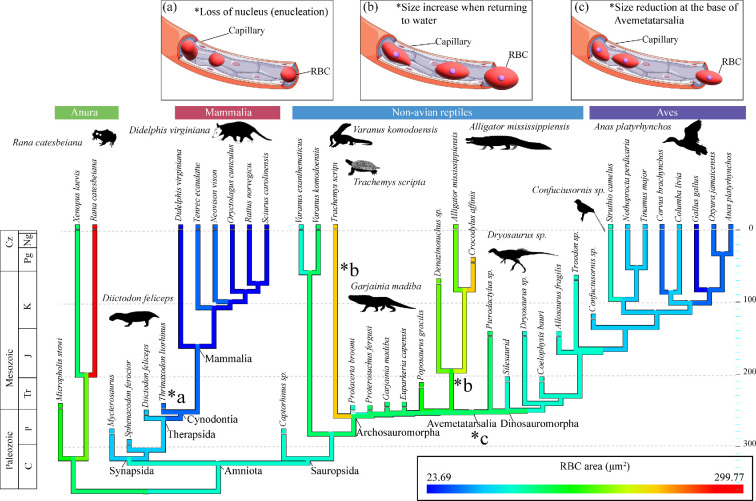
Time-calibrated heatmap phylogeny displaying changes in RBC_area_. Decreases in RBC_area_ occur at the base of Avemetatarsalia and continue in bird-line archosaurs. Meanwhile, RBC_area_ increases in Pseudosuchia, especially when they begin to secondarily adapt to the water. (a), (b) and (c) illustrate the morphology of the capillary and RBCs at the node of Cynodontia, Pseudosuchia, and Avemetatarsalia. Small RBCs are found throughout Synapsida, with a sharp decrease in area at Cynodontia and Mammalia (a), potentially representing enucleation. Increased RBC area occurs when pseudosuchians return to the water (b). A reduced RBC area is depicted by small, elliptical RBCs at the base of Avemetatarsalia (c). Credit for Phylopic silhouettes goes to: Smokeybjb (*Diictodon feliceps*), Gabriela Palomo-Munoz (*Didelphis virginiana, Glyptemys insculpta*), Mark Witton (*Garjainia*), Armin Reindl (Caimaninae), Scott Hartman, modified by T. Michael Keesey (*Eocuror parvus*) and Matt Martyniuk (*Protopteryx fengningensis*); credit for a silhouette not on Phylopic goes to sore88 (*Varanus komodoensis*).

Relationships were assessed between RBC size (RBC_width_, RBC_area_, RBC_length_) and bone histometry from thin sections of limb bones and blood smears from 20 extant tetrapods (expanded from the original Huttenlocker & Farmer dataset; [10]). Our multivariate models incorporated three log-transformed histometric variables: osteocyte lacuna volume (Osteo_volume_), vascular canal harmonic mean diameter (Can_harmean_) and canal minimum calibre (Can_min_), as well as RBC_width_, RBC_area_ and RBC_length_ as dependent variables. Histological data were analysed in R 1.4.1103 [18] to construct phylogenetic eigenvector maps (PEM) using R package MPSEM [19] to predict RBC sizes in the extant and extinct species. In PEM, a set of descriptor variables is built by converting a phylogenetic tree into an influence matrix that incorporates topology and edge length information, from which eigenvectors can be selected through a forward stepwise selection procedure to maximize a linear regression model fit with a response variable. Extracted eigenfunctions predict potential quantitative patterns based on input data from the phylogeny itself, depicting variations within taxa represented in a tree for which crucial data are missing [19,20]. We extracted phylogenetic eigenvectors and assessed their power as co-predictors with Osteo_volume_, Can_harmean_ and Can_min_ to predict RBC_area_, RBC_width_ and RBC_length_. Here, the model incorporating lnCan_min_ + phylogeny was selected as the best fit (*R*^2^ = 0.77, *p* = 1.569 × 10^−06^ using a Holm–Sidak Correction model (see table 1) for reporting the RBC_area_ values in figures 1 and 2. As suggested in our previous study [10], histomorphology and phylogeny serve as strong co-predictors of RBC size in tetrapods.

**Table 1.** Four models were used to calculate the estimated RBC values. lnCan_min_ + phylogeny was selected due to having the best fit (*R*^2^ = 0.77, *p* = 1.569 × 10^−06^ using a Holm–Sidak correction model.

model selection/co-predictor	*R*^2^ value	*p*-value
lnOsteo_volume_ + phylogeny	0.7532168	1.049e^−05^
lnCanal_harmean_ + phylogeny	0.6416886	6.319e^−05^
InCanal_min_ + phylogeny	0.7680194	1.569e^−06^
phylogeny	0.5476686	0.0001155

Our integrated analysis of histology and phylogenetic eigenvectors, incorporating bone histological data from 20 additional fossil taxa (40 extant and fossil taxa in total), suggests divergent patterns of RBC size evolution in bird-line (Avemetatarsalia) and croc-line (Pseudosuchia) archosaurs (figure 1). Whereas the early-diverging avemetatarsalians exhibited relatively small predicted RBC_area_ (less than 90 µm^2^), pseudosuchians exhibit large RBC_area_ in both terrestrial Triassic species (e.g. *Poposaurus gracilis*: RBC_area_ = 138.1 µm^2^) and later-evolving aquatic species (*Crocodylus affinis:* RBC_area_ = 124.1 µm^2^; *Denazinosuchus*: RBC_area_ = 116.4 µm^2^), but were consistently large in the aquatic species. This includes the secondarily aquatic crocodylian, *Alligator mississippiensis* (RBC_area_ = 159 µm^2^), and the sampled testudine, *Trachemys scripta* (RBC_area_ = 205 µm^2^).

## 3. Discussion

### (a) Large RBC size and semi-aquatic adaptations in crocodile-line archosaurs

Since the publication of the study by Huttenlocker & Farmer [10], other studies have suggested links between energetic lifestyle and RBC size in modern archosaur clades, especially in birds [8]. As in some semi-aquatic birds [8], increased RBC sizes in modern crocodilians potentially represent a relaxed evolutionary constraint on oxygen delivery due to the lower energetic demands and cost of transport in aquatic locomotion compared with their terrestrial ancestral state [1,14].

Furthermore, additional musculoskeletal, audio-vestibular and cardiovascular adaptations of extant crocodilians were secondarily acquired deep within Pseudosuchia as members returned to an aquatic lifestyle (e.g. dorsoventrally flattened skull, muscular tail, secondary palate, dorsally located nares, dorsally located external ear with a strong valvular flap, the foramen of Panizza, the cog-toothed valve; [1,2,21–24]). The benefits of these adaptations range from aiding in aquatic locomotion to reducing systemic arterial oxygen tension by creating cardiac shunts—lowering metabolic rate to facilitate a sit-and-wait ambush hunting style [1]. Increases in RBC size as a response to a secondarily aquatic transition in Pseudosuchia [1,8,25,26] are consistent with the pattern seen in a large range of other lineages, such as marine mammals [27].

An important finding of this study is that large RBC sizes were present in the extinct terrestrial pseudosuchian *Poposaurus gracilis*, which suggests a lower maximum aerobic capacity compared with their forebears. This could indicate that a lower aerobic capacity and reduced activity metabolism may have already been present in some pseudosuchians before secondary adaptation to an aquatic environment. This reveals an uncoupling between rapid bone growth (evidenced by woven-parallel complex) within poposauroids and rates of maximal aerobic capacity. Extant crocodilians have slow bone growth that comprises primarily parallel-fibred or lamellar bone tissue types [28]. It is important to further investigate which large-scale physiological shifts within early-branching pseudosuchians were already present before secondarily returning to the water. This is because both lower exercise aerobic capacities and slower growth strategies had already been acquired among terrestrial crocodylomorphs across the Triassic–Jurassic boundary, with ectothermy probably being a synapomorphy of Metasuchia [14,29]. Additionally, it is noteworthy to consider that physiological adaptations for low O_2_, aquatic environments may have led to the canalization of large RBC sizes in secondarily aquatic non-avian reptiles. The developmental burden of an inherited, limited toolkit might have slowed their re-diversification and limited their ability to reoccupy other high-energy terrestrial or marine niches following mass extinction events.

Furthermore, it is important to note that metabolic strategies among extant tetrapods disprove the hypothesis that endothermy and ectothermy form a dichotomy. It is possible for an ectotherm such as the teiid lizard *Salvator merianae* to generate heat and retain it during the reproductive period to raise body temperature significantly above the ambient, the very definition of endothermy. Meanwhile, endotherms such as bats and hummingbirds, which have the highest rates of aerobic exercise capacities found in endotherms and among the smallest RBCs, do not generate sufficient heat to maintain body temperature significantly above ambient temperature during torpor [30,31]. For every lineage, selection on the energetic costs of endothermy is always entangled with resource availability, whether the resource is oxygen (such as deep in a burrow), food, water or the risk of predation while seeking resources. Most studies show that ectothermy is an advantageous strategy in environments where resources are scarce or where they fluctuate unpredictably [32]. While it has been suggested that becoming secondarily semi-aquatic was a selective pressure for ectothermy in crocodylians [33], there are examples of endothermy being retained in secondarily aquatic and semi-aquatic mammals and birds, and even evolving in some sea turtles [34]. It is therefore incumbent upon this hypothesis to explain why the crocodilian lineage differed from this broader pattern, in which another likely driver was a trade-off between elevated metabolism and resource availability.

### (b) Energetics in stem archosaurs and bird-line archosaurs

The success of Archosauromorpha following the Permo-Triassic mass extinction is marked by intense physiological and morphological experimentation [12]. Our model shows varying RBC sizes across sampled archosauromorphs. For example, the South African non-archosauriform archosauromorph, *Prolacerta broomi*, exhibits relatively small RBC sizes (RBC_area_ = 78.61 µm^2^) compared with the archosauriforms *Euparkeria capensis* (RBC_area_ = 106.93 µm^2^), *Proterosuchus fergusi* (RBC_area_ = 124.57 µm^2^) and *Garjainia madiba* (RBC_area_ = 133.98 µm^2^). This suggests *Prolacerta* probably had a high maximal aerobic capacity. These data are corroborated by the presence of a rapid, continuous growth strategy, enabling *Prolacerta* to reach sexual maturity quickly [35,36], along with a high predicted mass-specific resting metabolic rate [9]. In addition, endothermy confers on lineages the ability to shorten the time between conception and sexual maturity, which can be critical for matching a reproductive bout to an ephemeral resource [37,38]. In addition, diminutive RBC sizes are also found in hypoxia-adapted species (e.g. high-altitude birds, burrowing mammals) [39–41]. While its life history remains unknown, *Prolacerta* could have been an active burrower or an occupant of already-created burrows, as inferred in small subterranean Triassic cynodonts and some non-archosauriform archosauromorphs [42,43]. These characteristics (i.e. small RBC sizes, high mass-specific resting metabolic rate and rapid growth strategy) might have allowed *Prolacerta* to persist and adapt to the monsoonal-type torrential rainfall and extreme drought that occurred in the Early Triassic Karoo Basin [35,44–46].

By the Middle to Late Triassic, marked decreases in RBC size are found near the divergence of Avemetatarsalia, starting with the pterosaur *Pterodactylus* (RBC_area_ = 53.9 µm^2^) and a silesaurid dinosauromorph/potential early-diverging ornithischian dinosaur (RBC_area_ = 73 µm^2^). This suggests the earliest diverging bird-line archosaurs were capable of sustained, vigorous exercise, reflecting a high maximal aerobic capacity. These data coincide with shifts in posture (semi-erect to erect) in early bird-line archosaurs and the first occurrences of bipedalism in early dinosaurs, as well as increases in estimated stride lengths, leg length and average speed [12]. Furthermore, increased growth rates [47], elevated basal metabolic rates [9,48], the possible presence of pycnofibers [49] and the hypothesized evolution of an extremely thin blood–gas barrier [50] would have enabled early avemetatarsalians to occupy new niches, remain active year-round at high latitude and altitude [51,52], and to be resilient to a variety of climatic conditions. During the latter half of the Triassic, when avemetatarsalians were diversifying, huge fissure eruptions across what is now western North America created the Wrangellia basalts [53]. This escalated greenhouse gas emissions in the atmosphere, causing global warming and widespread ocean anoxia—the Carnian Pluvial Event [12,54]. As many as five cyclical pulses are thought to have occurred during approximately a 1 Myr interval from 233 to 232 Ma [55], resulting in constant switches between arid and humid conditions that triggered widespread ecological turnover [12,53,54,56]. Accordingly, selection on RBC size in avemetatarsalians may have been constrained by both a maximal aerobic capacity and/or adaptations for hypoxia caused by fluctuations in Triassic environmental conditions.

### (c) Origin of powered flight in archosaurs

Despite detailed anatomical reconstructions, opinions on the origin of powered flight and aerial capabilities of pterosaurs and Mesozoic birds have ranged from minimal suspected flight capabilities, to obligate gliders, long-distance soarers, to capable of short periods of powered flight [57–60]. A recent study on Mesozoic bird flight [61] identified *Confuciusornis sanctus* as being capable of prolonged powered flight mixed with alternating periods of high-efficiency gliding. The energetic cost would necessitate an increased aerobic capacity to allow for powered flapping and active aerial foraging. In this study, marked RBC size reductions are exhibited in the two major branches of bird-line archosaurs (Avemetatarsalia), which include *Pterodactylus* and *Confuciusornis*. These RBC values (*Pterodactylus*: RBC_area_ = 53.6 µm^2^; *Confuciusornis*: RBC_area_ = 68.9 µm^2^) overlap those of extant Neornithes (e.g. the common pigeon *Columba livia*: RBC_area_ = 69.4 µm^2^). At a minimum, this suggests *Pterodactylus* and *Confuciusornis* were capable of intense energetics and aerobic muscle activity or that these size reductions were a result of hypoxic conditions at high altitude, both of which would have been a prerequisite for prolonged powered flight, as suggested by previous studies [61].

## 4. Experimental procedures

### (a) Data collection

To assess the relationship between blood values and femoral bone histology, the extant tissue dataset of Huttenlocker & Farmer [10] was increased to 20 taxa with the addition of more volant and flightless birds (e.g. *Corvus*, *Struthio*) and several mammals (e.g. *Tenrec*). Some fossil taxa were added to include more extinct archosaurs, including a pterosaur (*Pterodactylus*), non-avian dinosaurs (e.g. *Dryosaurus*, *Coelophysis*), extinct crocodilians (*Denazinosuchus*) and a Mesozoic bird (*Confuciusornis*). The new fossil samples were provided by the Natural History Museum of Utah (*Denazinosuchus*, *Allosaurus*) and the Museum of the Rockies (all other non-*Allosaurus* avemetatarsalian specimens). The resulting dataset included histological measurements from 40 taxa in total (20 extant, 20 extinct). Values for RBC_width_ and RBC_area_ were measured in the extant species from blood smears (although blood values for *Struthio* and *Tinamus* were substituted from the literature; see electronic supplementary material). Bone histological traits, including minimum (Can_min_) and harmonic mean (Can_harmean_) canal calibre and osteocyte lacuna volume (Osteo_volume_), were measured from midshaft femur cross sections. All measurements were performed in ImageJ v. 1.47 [62] using captured image series around the bone circumference. These data were incorporated into a character matrix provided in the electronic supplementary material. The code used to run the analysis in R is also included in the electronic supplementary material.

### (b) Models and phylogenetic methods

From the character matrix, PEMs were constructed in the integrated development environment *R* [18]. Model estimation was performed using the R package *MPSEM* [19] v. 0.5-1, with additional functions from packages *evobiR* v. 1.1 [63], *Metrics* v. 0.1.4 [64], *ape* v. 5.8 [65], *MASS* v. 7.3 [66] and *maps* v. 3.4.2.1 [67], so that RBC sizes in the extant and extinct species could be retrodicted from the bone histological data and the phylogeny. We extracted phylogenetic eigenvectors as co-predictors with Osteo_volume_, Can_harmean_ and Can_min_ to construct four predictive models estimating RBC mean area. The phylogenetic tree was constructed in *Mesquite* v. 3.81 [68] and imported into *R* using *ape* [65].

## Data Availability

Electronic supplementary material includes both histometric and blood smear values, as well as the complete R code. These files can be found on Dryad at [69]. Supplementary material is available online [70].
